# Binasal Quadrantanopia: A Rare Visual Field Defect Poses Challenges in Terms of Lesion Localization

**DOI:** 10.7759/cureus.57911

**Published:** 2024-04-09

**Authors:** Yu-Han Wang, Chen Li, Wen-Ching Chen, Poyin Huang

**Affiliations:** 1 School of Medicine, College of Medicine, Kaohsiung Medical University, Kaohsiung, TWN; 2 Department of Neurology, Kaohsiung Medical University Hospital, Kaohsiung Medical University, Kaohsiung, TWN; 3 Department of Neurology, Kaohsiung Municipal Siaogang Hospital, Kaohsiung Medical University, Kaohsiung, TWN; 4 Neuroscience Research Center, Kaohsiung Medical University, Kaohsiung, TWN; 5 Dysphagia Functional Reconstructive Center, Kaohsiung Municipal Siaogang Hospital, Kaohsiung Medical University, Kaohsiung, TWN; 6 Multidisciplinary Swallowing Center, Kaohsiung Municipal Siaogang Hospital, Kaohsiung Medical University, Kaohsiung, TWN; 7 Department of Neurology, Faculty of Medicine, College of Medicine, Kaohsiung Medical University, Kaohsiung, TWN

**Keywords:** neuro-ophthalmology, anterior ischemic optic neuropathy, lesion localization, visual field defect, quadrantanopia

## Abstract

Binasal quadrantanopia is a rare type of visual field defect characteristic of vision loss in either the upper or lower quadrants of both nasal visual fields. The affected individuals often exhibit impairments in peripheral vision, leading to difficulties in various daily activities such as navigation, object recognition, and hazard avoidance. The consequences of binasal quadrantanopia can be profound, affecting the individual's quality of life and functional independence. However, due to its atypical presentation and overlapping clinical features with other visual field defects, accurately pinpointing the lesion's precise location for further management becomes a complex task. Here, we present an unusual case of binasal quadrantanopia caused by bilateral anterior ischemic optic neuropathy (AION) and aim to explore the unique characteristics, etiology, and diagnostic approaches associated with binasal quadrantanopia, shedding light on the challenges encountered in lesion localization.

## Introduction

Binasal quadrantanopia is an exceedingly infrequent visual field defect. The scarcity of documented cases of binasal quadrantanopia in scientific literature not only highlights its infrequency but also underscores the difficulties in diagnosing and comprehending its various causative factors. Specifically, identifying the exact site of the lesion responsible for this specific visual field defect remains a challenge.

Due to its distinct and symmetrical nature, binasal quadrantic defects are often misattributed to brain lesions [1]; however, their origin can be traced to a spectrum of underlying factors or pathologies affecting the optic nerves [1-2]. This complexity emphasizes the importance of comprehensive examination in understanding and effectively managing such cases.

Our case is particularly valuable due to the intricacies involved in identifying the exact lesion responsible for this specific visual field defect. It significantly contributes to the discussion and comprehension of this rare condition.

## Case presentation

A 71-year-old man, who is a known case of hyperlipidemia, hyperuricemia, and chronic kidney disease (CKD) stage 3, presented with an acute onset of painless right eye blurred vision with a stationary course for 2 weeks. He denied loss of consciousness, fever, chills, headache, vertigo, tinnitus, hearing impairment, diplopia, facial palsy, dysphagia, dysarthria, choking, nausea, vomiting, unsteady gait, focal weakness, and focal numbness.

**Figure 1:**
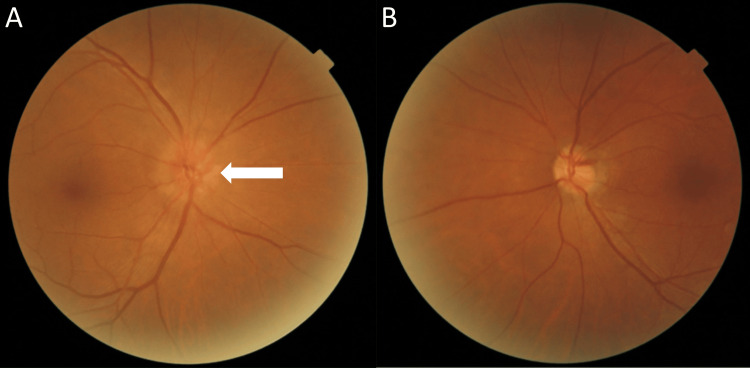
Dilated fundus examination using an ophthalmoscope The right (A) and left (B) retina, macula, and optic nerve were visualized with pupil dilation. Fundoscopy revealed no break or hemorrhages in the bilateral retina but revealed right optic disc edema (white arrow).

At presentation, visual acuities were 20/20, bilaterally. No relative afferent pupillary defect (RAPD) was detected in both eyes. Fundoscopy revealed a swollen right optic disc without hemorrhages or a break in the bilateral retina (Figure 1).

**Figure 2:**
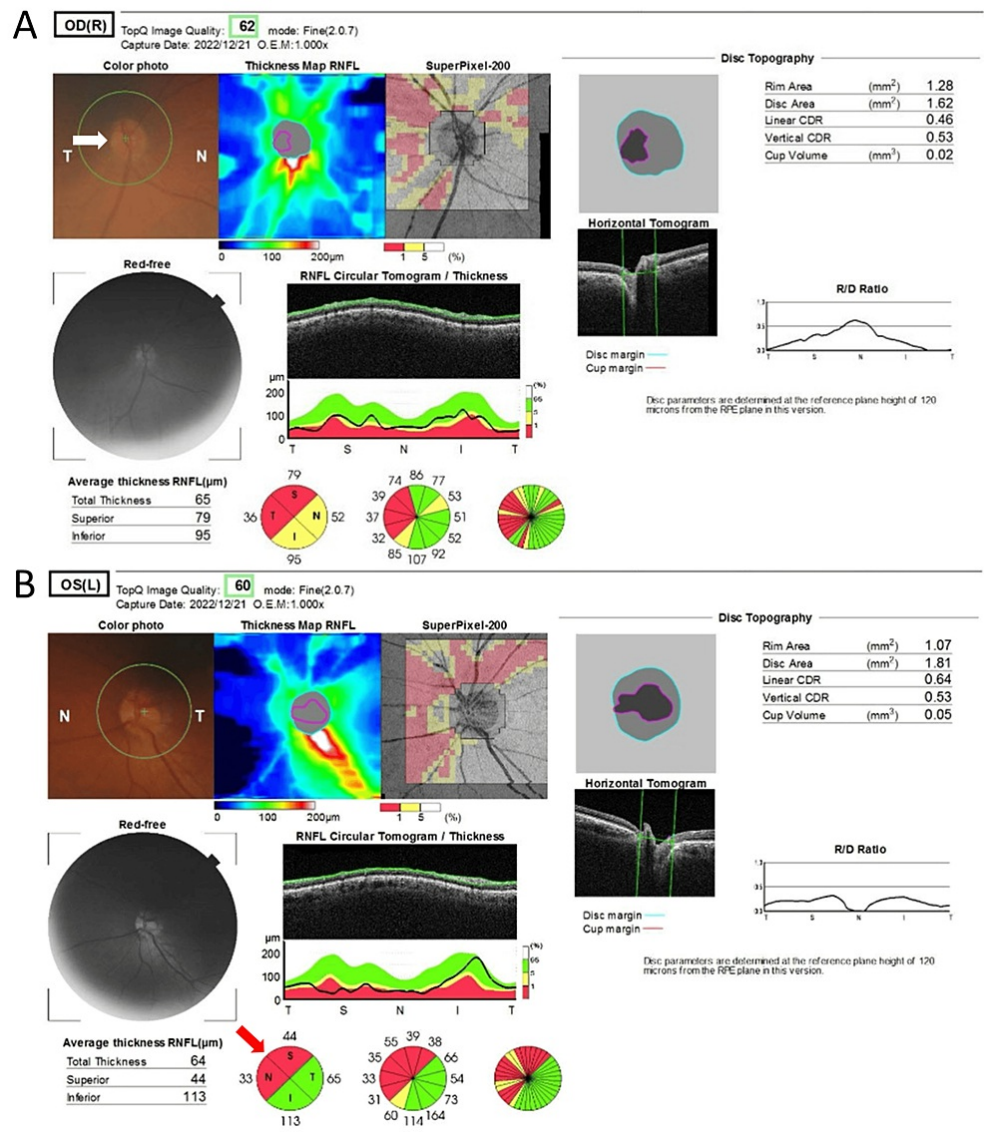
Retinal photographs and thickness evaluation using optical coherence tomography Retinal nerve fiber layer defects were observed in both eyes. Optical coherence tomography showed (A) right optic disc edema (white arrow) and (B) thinning of the left retinal nerve fiber layer in the nasal superior aspect (red arrow).

Optical coherence tomography (OCT) revealed right optic disc edema and left retinal nerve fiber layer (RNFL) thinning at the superior and nasal quadrants (Figure 2).

**Figure 3:**
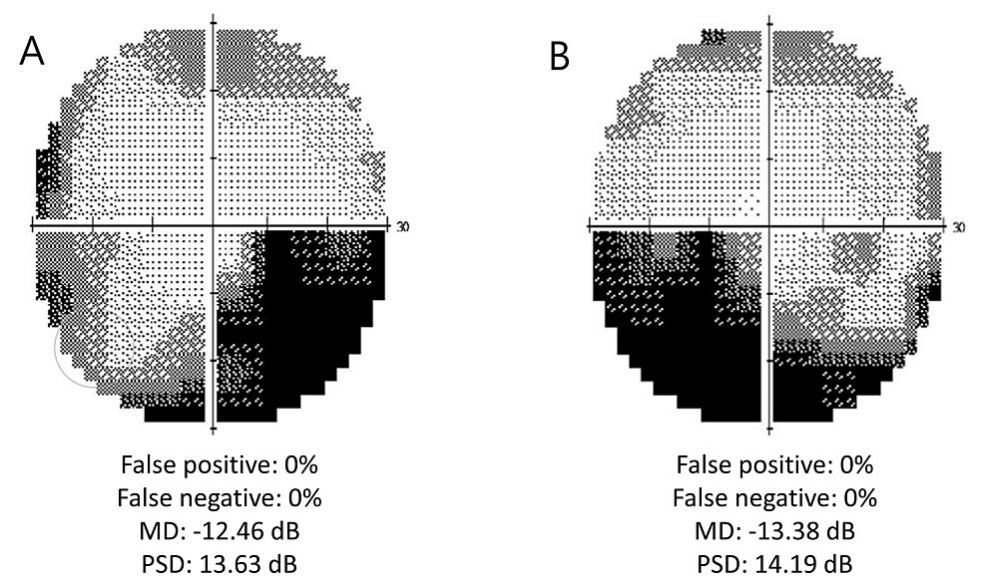
The results of a visual field examination using a Humphrey field analyzer Humphrey Visual Fields (30-2) showing right eye (A) and left eye (B) visual field defects in the nasal inferior aspect MD = mean deviation; PSD = pattern standard deviation

Humphrey Visual Field (HVF) showed a right nasal-lower quadrantal to altitudinal defect and a left nasal-lower quadrantal defect (Figure 3).

**Figure 4:**
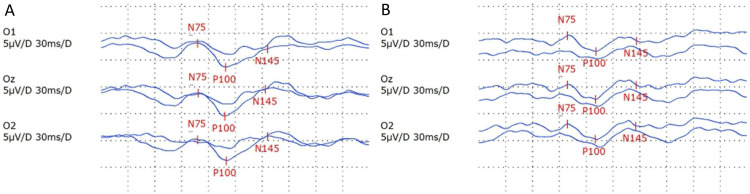
Waveforms of VEP to stimulation The N75, P100, and N145 waves were labeled. Normally, the P100 peak appears around 100-120 milliseconds after the visual stimulus is presented. In this case, both the right (A) and left (B) eye show a delay in the P100 peak, indicating a slower transmission of visual information from the retina to the visual cortex. VEP: visual-evoked potential

For further investigations of binasal lower quadrantanopia, the patient was admitted and was co-managed by the neuro-medical team. The visual-evoked potential (VEP) test showed a prolongation of P100 latency in both eyes, as indicated in Table 1 and Figure 4.

**Table 1:** Right and left eye VEP 3 channel measurements VEP: visual-evoked potential

VEP 3 Channel								
			Latency			Amplitude		
			Right	Left	Interocular differences	Right	Left	Interocular differences
			ms	ms	ms	μV	μV	μV
Mean	O1	N75	108	99.4	8.6	0.66	0.22	0.44
		P100	139	131	8.0	-2.7	-1.94	0.76
		N145	186	176	10.0	-0.16	0.036	0.20
	Oz	N75	109	99.4	9.6	-0.015	-0.080	0.065
		P100	138	130	8.0	-3.1	-2.4	0.70
		N145	184	176	8.0	0.77	0.28	0.49
	O2	N75	108	99.4	8.6	-0.056	0.11	0.17
		P100	140	130	10.0	-2.5	-2.2	0.30
		N145	186	175	11.0	0.97	0.36	0.61

**Figure 5:**
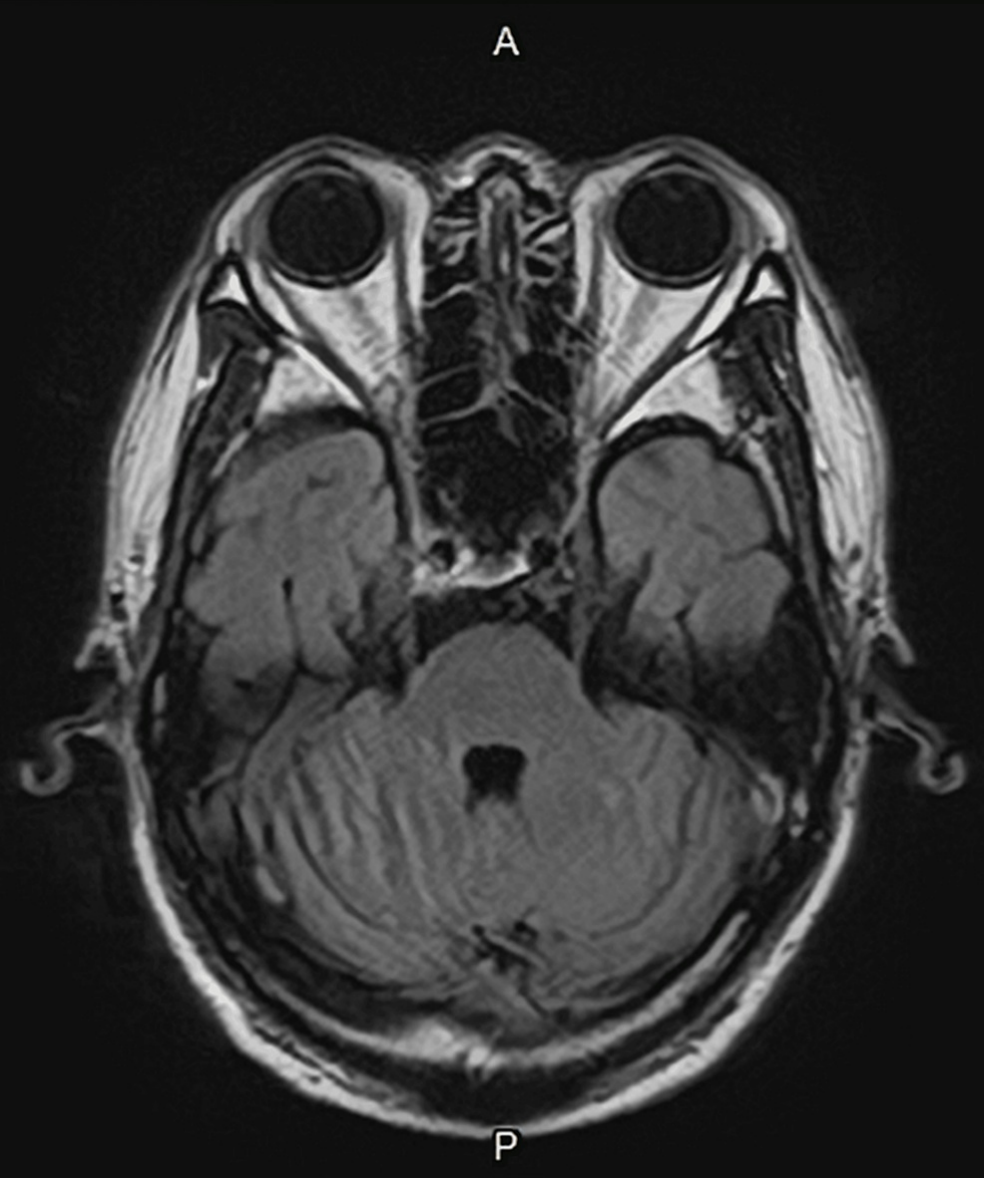
Contrast-enhanced magnetic resonance imaging of the brain and orbits. T2-weighted-fluid-attenuated inversion recovery revealed no inflammation or occupying lesion along the visual pathway. A = anterior; P = posterior

This prolongation may be attributed to either bilateral prechiasmatic lesions and/or retrochiasmatic lesions. To ascertain the precise location of the lesion, contrast-enhanced magnetic resonance imaging (MRI) of the brain and orbits was conducted. However, no profound signs of inflammatory changes or compression lesions were observed in the imaging study (Figure 5).

According to his clinical manifestations and the examination results of the fundus and brain, while exclusive of other possible diseases that might affect the visual pathway, a diagnosis of bilateral anterior ischemic optic neuropathy (AION) was made. The patient was given antiplatelets (aspirin 100 mg/day) and antihyperlipidemic (atorvastatin 40 mg/day) to control risk factors along with vasodilators (nicergoline 20 mg/day) that improved the arterial blood flow to the brain. After treatment, the patient's neurologic signs and symptoms remained stationary.

## Discussion

The visual pathway follows a specific projection rule and forms a spatial pattern of cortical excitation. Starting at the retina, the optic nerve fibers then partially decussate at the optic chiasm. Stimulated fibers from the contralateral visual field reach the lateral geniculate nuclei via optic tracts and continue to the cortex through optic radiations. Lesions along this pathway can cause specific visual field defects. While retro-chiasmal lesions typically result in homonymous deficits, binasal quadrantanopia is likely with pre-chiasmal or chiasmatic lesions.

Accurately localizing the precise lesion site can be challenging due to several factors. First, from a neuro-ophthalmic standpoint, this atypical visual field defect pattern is difficult to explain through the neuroanatomy of the afferent visual pathway. Second, the rarity of binasal quadrantanopia makes it less well-known among clinicians, leading to potential misdiagnosis or delayed recognition. Moreover, the presentation of binasal quadrantanopia may overlap with other visual field defects, such as bitemporal hemianopia or homonymous defects, making differential diagnosis critical.

The underlying etiology of binasal quadrantanopia can vary, including compressive lesions, inflammatory processes, vascular abnormalities, and congenital anomalies, which further complicates the process of localizing the lesion [3-5]. In the presented case, combining the history with thorough examination results, the ophthalmologist ruled out the possibility of intracranial causes and finally came to the diagnosis of bilateral AION. The typical presentation of AION is a sudden and painless monocular deterioration of vision, accompanied by decreased visual acuity, which can vary from 20/20 to no light perception [6]. Additionally, there may be visual field defects, with a combination of a relative inferior altitudinal defect and an absolute inferior nasal defect being the most common presentation [7].

The accurate diagnosis of binasal quadrantanopia requires comprehensive diagnostic strategies, including a detailed medical history, ophthalmic examination, perimetry testing, neuroimaging techniques, such as MRI or computed tomography (CT) scans, and collaborative assessment involving ophthalmologists, neurologists, and radiologists. Further research and advancements in neuroimaging techniques, such as diffusion tensor imaging or functional MRI, also provide valuable insights into the precise anatomical and functional characteristics of the lesion.

## Conclusions

Binasal quadrantanopia, while relatively uncommon in clinical practice, presents significant challenges in accurately localizing lesions within the neuroanatomical framework. The rarity of this condition, coupled with its intricate presentation, underscores the importance of further elucidating its underlying mechanisms and localization patterns. By addressing the complexities associated with lesion localization in binasal quadrantanopia, we aim to contribute to the knowledge base, facilitating early recognition and appropriate management of this visually debilitating condition.
